# Supplementary material for: Prepared to Accompany the End of Life during Pandemics in Nursing Homes
Source: J Clin Med. 2022 Oct 14;11(20):6075. doi: 10.3390/jcm11206075 (PMC9604931; doi:10.3390/jcm11206075)
Supplement: Supplementary file 1 [file jcm-11-06075-s001.zip › jcm-1917297-supplementary.pdf]

Supplementary Table S1: content of the PallPan recommendations according to Gauder [11] and selected recommendations for nursing homes (x)

| Section | PallPan recommendations |
|---|---|
| **1—Supporting patients and relatives** | |
| 1a—Ensuring palliative care for infected and non-infected individuals | |
| | 1: Provision of best possible care for infected and non-infected severely ill and dying patients and their relatives (x)<br><br>2: Integration of the expertise and resources of specialist palliative care in the treatment process (x)<br><br>3: Recognition of the risk of loneliness of infected severely ill and dying people and avoidance of stigma and undersupply of patients (x)<br><br>4: Offering palliative care when making prioritization decisions (x)<br><br>5: Consideration of palliative care patients' concerns in prioritization concepts (x) |
| 1b—Documenting and respecting patient's will | |
| | 6: Offering early conversations about goals of care and treatment preferences for severely ill patients or at risk for a severe infection course (x)<br><br>7: Documenting and respecting patient's will (x) |
| 1c—Ensuring visits and proximity between patients and relatives | |
| | 8: Balancing the individual needs of patients with the protection of the public from infection (x)<br><br>9: Enabling visiting and company of relatives (x)<br><br>10: Creation of separate regulations for patients receiving palliative care when establishing contact restrictions (x)<br><br>11: In case of general visiting restrictions, creation of separate visiting concepts for severely ill and dying patients and their relatives (x)<br><br>12: Provision of sufficient protective equipment and personnel for visits by relatives (x)<br><br>13: In case of visiting restrictions, offering of intensified company by staff, chaplains, and volunteers (x)<br><br>14: Provision of means of communication (x) |
| 1d—Ensuring exchange of information and communication between professional carers and relatives | |
| | 15: Regular contacting of relatives by professional carers (x)<br><br>16: Informing relatives and patients about regulations (x) |
| 1e—Enabling farewell after death | |
| | 17: Enabling farewells to be said to the deceased (x) |

| | |
|---|---|
| | 18: Enabling participation in funerals<br>19: Offering bereavement support(x) |
| **2—Supporting staff** | |
| | 20: Ensuring infection prevention for staff (x)<br>21: Informing staff regularly about the pandemic situation and current regulations (x)<br>22: Encouraging staff for regular feedback to responsible persons about their problems and needs (x)<br>23: Training of staff in the treatment and care of the seriously ill and dying (x)<br>24: Establishing low-threshold support for staff (x)<br>25: Establishing opportunities for interdisciplinary and multi-professional exchange (x) |
| **3—Supporting and maintaining structures and provision of palliative care** | |
| 3a—Maintaining palliative care services | |
| | 26: Maintaining existing generalist and specialist palliative care services and structures<br>27: Exploring the need for expansion or new creation of palliative care provision<br>28: Providing preconditions and the framework for digital communication (x) |
| 3b—Integrating palliative care aspects into federal and state governments and local administrations (incl. crisis teams and pandemic plans) | |
| | 29: Providing additional short-term financial resources for the care of severely ill and dying people<br>30: Naming and including palliative care experts in the development and implementation of pandemic plans<br>31: Naming and including palliative care experts in crisis teams<br>32: Networking of palliative and hospice services during the pandemic<br>33: Naming contact persons responsible for palliative care issues in public authorities |
| | |

Gauder, S., Pralong, A., Rémi, C., Hodiamont, F., Klinger, I., Heckel, M., Simon, S. T., & Bausewein, C. (2022). Development of a national strategy with recommendations for the care of seriously ill and dying people and their relatives in pandemics: A modified Delphi study. *Palliat Med*, *36*(8), 1285-1295. https://doi.org/10.1177/02692163221114536
